# Emotions in Group Sports: A Narrative Review From a Social Identity Perspective

**DOI:** 10.3389/fpsyg.2019.00666

**Published:** 2019-03-29

**Authors:** Mickael Campo, Diane M. Mackie, Xavier Sanchez

**Affiliations:** ^1^Université Bourgogne Franche-Comté, Laboratoire Psy-DREPI: Psychologie – Dynamiques Relationnelles Et Processus Identitaires (EA-7458), Dijon, France; ^2^Psychological and Brain Sciences, University of California, Santa Barbara, Santa Barbara, CA, United States; ^3^Center of Research on Welfare, Health and Sport, Halmstad University, Halmstad, Sweden

**Keywords:** group-based emotions, group belonging, intergroup relations, intergroup emotions, social identity

## Abstract

Recently, novel lines of research have developed to study the influence of identity processes in sport-related behaviors. Yet, whereas emotions in sport are the result of a complex psychosocial process, little attention has been paid to examining the mechanisms that underlie how group membership influences athletes’ emotional experiences. The present narrative review aims at complementing the comprehensive review produced by Rees et al. (2015) on social identity in sport by reporting specific work on *identity-based emotions* in sport. To that end, we firstly overview the different terminology currently used in the field of emotions in groups to clarify the distinct nature of emotions that result from an individual’s social identity. Secondly, we discuss key concepts of social identity to better understand the mechanisms underlying identity-based emotions. Thirdly, we address existing knowledge on identity-based emotions in sport. We close the present narrative review by suggesting future research perspectives based on existing meta-theories of social identity. Evidence from the social psychology literature is discussed alongside existing works from the sport literature to propose a crucial theoretical approach to better understand emotions in sport.

## Introduction

Emotions’ omnipresence in human life has prompted scientists across various disciplines to investigate their role to better understand their influence on performance, health, and wellbeing. Achievement contexts, and especially those such as sport, are of particular interest when it comes to studying frequency, intensity, and balance of emotional experiences (Lazarus, 2000). Indeed, competitive athletes are continuously under performance outcome pressures, and may feel happy or sad as they win or lose, angry toward an opponent who is cheating, and pride, excitement, or even surprise if they score a winning point against their biggest opponent. Traditionally, scientific research in the field of sport psychology was developed within a rather reductionist view of emotional processes, by mainly focusing on the individual rather than considering the social-self. However, emotions are social by nature and have an adaptive role in humans’ functioning (Lazarus, 1999) in their social environment (Fischer and Manstead, 2008) to such extent they are now recognized as “an ubiquitous aspect of interaction between groups” (Mackie and Smith, 2018, p. 1). Accordingly, a great deal of literature in the field of emotions recognizes the different social functions of emotions. In synthesis, they offer information about a social event, help prepare people to respond to problems and opportunities, organize interpersonal relationships, and influence others (Keltner and Haidt, 1999; Niedenthal and Brauer, 2012). At the group-level of analysis, emotions help individuals define group boundaries, as well as help them resolve group-related problems (Keltner and Haidt, 1999; Niedenthal and Brauer, 2012), react to group threatening events (Smith and Mackie, 2008), and maintain group vitality (Niedenthal and Brauer, 2012).

In sport, the social dimension of emotions has been demonstrated through the myriad of different recognized sport-specific emotional triggers. For instance, Nicholls et al. (2009) showed that “receiving criticism from a coach or parent,” “watching an opponent perform,” “being distracted by viewers,” “receiving a referee’s warning,” and “watching an opponent cheating” represented five of the top-ten most common stressors faced by rugby players, which highlight that athletes’ emotions are continuously influenced by the social context. In that sense, recent work in sport psychology has also emphasized that emotions may be directly regulated by others (i.e., interpersonal emotion regulation; Friesen et al., 2013; Tamminen et al., 2016; Campo et al., 2017) as well as collectively (i.e., communal coping; Leprince et al., 2018). However, while athletes’ social environment may directly influence their emotions, other more indirect social mechanisms seem also to play a role in the emotional process. For instance, why do players feel angry toward opponents, just because they are opponents? Accordingly, different authors have recently called to consider the social-self in the study of emotions in the context of competitive sport (e.g., Campo et al., 2012, 2017, 2018; Tamminen et al., 2016). It should be assumed that emotions in sport, especially because sport is almost always practiced in groups, are the result of a complex psychosocial process. However, it is rather surprising how little attention has been paid to studying the mechanisms that underlie how group membership influences an athlete’s emotional experience (Terry et al., 2000).

Emotions in groups have been extensively discussed in the social psychology literature, notably within the scope of social identity theories. Such frameworks are currently of a growing interest in the field of sport, as evidenced by the recent literature (e.g., Rees et al., 2015) and the organization of the first international conference of social identity in sport in 2017 (ICSIS, Leuven, Belgium). However, in line with the historical tradition of social identity theoretical theories, it could be noted that none of the communications presented at the 2017 ICSIS reported investigations of emotions at the group level. In this vein, Rees et al. (2015) also recently provided a comprehensive review of literature in social identity within the field of sport psychology. The authors reported works that examined the influence of social identity on sport-related behaviors, but did not focus as closely on the influence of identity processes on emotions.

Therefore, in light of the importance of emotions in the field of performance (Hanin, 2007, 2016; Hanin et al., 2016), the present narrative review complements Rees et al. (2015) work by addressing literature examining emotions at the group level. In particular, the current paper focuses on the effects of group belonginess on athletes’ emotions. To facilitate understanding, our paper is organized into four main sections. The first section clarifies terms currently used in the field of emotions in groups. Here we explain the main theoretical precepts associated with the different terms to make clear the distinct nature of emotions that result from an individual’s social identity, which are of interest in the current paper. The second section overviews the key concepts of social identity (for further details, see Rees et al., 2015) and the mechanisms underlying identity-based emotions. The third section reviews the existing literature about identity-based emotions in sport, while the last part of this narrative review is devoted to proposing future research perspectives on the basis of existing meta-theories of social identity.

## What Does Identity-Based Emotions Actually Mean? a Theoretical-Based Clarification of Terminologies

Literature examining emotions at the group level has suffered from a lack of clear terminology. Such terms – sometimes for similar concepts – include *collective emotions* (e.g., Doosje et al., 1998), *group emotions* (e.g., Parkinson et al., 2005), *vicarious emotions* (e.g., Lickel et al., 2005), *intergroup emotions* (e.g., Mackie et al., 2000; Smith et al., 2007), or *group-based emotions* (e.g., Kuppens and Yzerbyt, 2012; Mackie and Smith, 2018). Similarly, this is found in the sport literature, including *group-based emotions* (e.g., Tamminen et al., 2016; Campo et al., 2019b), *collective emotion* (Tamminen et al., 2016; Sullivan, 2018), *collective mood* (Totterdell, 2000), *group emotions* (Hyun-Woo and Youkyoum, 2013), and *team-referent emotions* (Campo et al., 2019b).

Although broadly associated to the topic of ‘emotions in groups,’ these terms refer to different concepts. Shortly, collective emotions (Sullivan, 2018) and collective mood (Totterdell, 2000) both echo other semantics in social psychology such as group or shared emotions, and mainly refer to a psychological phenomenon called emotional contagion. In sport competition, Totterdell (2000) showed, for the first time, the existence of emotional linkage across cricketers of the same team. Also, Moll et al. (2010) highlighted that the transference of individual positive emotions to teammates following a successful penalty shootout served a direct purpose in enhancing future team performance. Supporting the notion of emotional climate (Niedenthal and Brauer, 2012) and that of emotional similarity (Schachter, 1959), collective emotions in sport may be understood as synchronous emotional response among several individuals (athletes, coaches, and supporters) facing a shared event (Barsade, 2002; von Scheve and Ismer, 2013; Tamminen et al., 2016). In contrast, team-referent emotions is a term built from the theoretical approach of social appraisal, which refers to the individuals’ perception of others’ emotions (Manstead and Fischer, 2001; Bruder et al., 2014). Particularly, team-referent emotions in sport have been recently defined as the way players appraise their team’s emotions as an entity (Campo et al., 2019b).

While different in essence, collective emotions (i.e., catching others’ emotions) or team-referent emotions (i.e., appraising team’s emotions) underlie an influence of others’ emotions. Everyday examples of this influence abound in the sport setting. For instance, some evidence showed that sports fans’ emotions varied significantly with their team’s outcomes (e.g., Cialdini et al., 1976; Jones et al., 2012; Sullivan, 2018). Other examples include the shared sadness in players on a team who have just failed to score (Moll et al., 2010), or the shared panic among players in the last minutes of a game (Bar-Eli and Tractinsky, 2000). Fransen et al. (2014) also showed contagion between captains and their partners ultimately influencing performance. It is recognized that shared emotions lead to tighter bonds (Manstead and Fischer, 2001; Peters and Kashima, 2007) and group identification (Kessler and Hollbach, 2005; Smith et al., 2007). However, it is also assumed that feeling emotions influenced by others’ emotions is different from experiencing emotions as a group member, and/or on behalf of a group to which the person belongs (Totterdell, 2000).

This latter mechanism refers to the notions of group-based emotions (e.g., Kuppens and Yzerbyt, 2012; Mackie and Smith, 2018) or intergroup emotions (e.g., Mackie et al., 2000; Smith et al., 2007). Whereas some conceptual nuances may differentiate them theoretically (see Yzerbyt et al., 2006, for a discussion), group-based and intergroup emotions can be subsumed into the notion of *identity-based emotions*. Defined as “emotions that arise when people identify with a social group and respond emotionally to events or objects that impinge on the group” (Smith and Mackie, 2008, p. 428), identity-based emotions are influenced by group membership and are, therefore, rooted in the person’s social identity (Kuppens and Yzerbyt, 2012).

## From Social Identity to Identity-Based Emotions. Key Theoretical Concepts

Social identity is considered as a key theoretical framework for understanding group functioning (Brown, 2000; Haslam, 2004; Rees et al., 2015; Thomas et al., 2017). In general, social identity refers to that part of intergroup relations that explains individuals’ behaviors – as members of an ingroup – in the presence of an outgroup (whether such presence is real, expected or imagined). Social identity is defined as a motivated cognitive mechanism that influences individuals’ perceptions of themselves based on their knowledge of their membership in a (social) group (Tajfel, 1978; Haslam, 2004). Thus, individuals may attach a value of an emotional significance to their group, which results in some degree of group identification, that then guides their behaviors (Tajfel, 1978; Tajfel and Turner, 1979).

In this sense, social categorisation is considered as a key process of social identity. Social categorization allows people to reduce the complexity of the actual social environment by understanding it through the spectrum of categories that cluster individuals sharing key attributes together, leading at times to stereotypes (including in sport, e.g., Martiny et al., 2015). In a situation of social comparison (such as the prototypical example of sport competition), social categorisation plus the level of group identification may contribute to ingroup bias, a preference for the ingroup over outgroups. More particularly, a central assumption of Social Identity Theory (Tajfel and Turner, 1979) is that individuals are motivated by a desire to maintain a positive view of the self. In order to protect their self-esteem, then, group members try to attain and maintain a positive social identity by promoting the ingroup and discriminating against members of outgroups. Although the self-esteem hypothesis has been criticized (Brown, 2000), the general influence of ingroup bias on behavior is widely established – including in sport where, for example, fans routinely denigrate other teams’ fans (e.g., Leach et al., 2003).

Moreover, it is recognized that a person may have multiple social identities associated with his or her simultaneous membership in multiple different groups or social categories (Turner et al., 1987). For illustration in the context of sport, a rugby player may react as an athlete, a rugby player, or as a member of his or her team. Also, research on social identity has demonstrated that individuals may choose among several social identities to protect or value their self-esteem (Tajfel, 1978; Turner et al., 1987; Mackie and Smith, 1998; Haslam, 2004). More particularly, when one group becomes less positive for its members, individuals may, under certain circumstances, abandon that social identity and migrate to another one. This identity strategy, also called *social mobility*, could be used, for instance, when one’s current team is accruing defeats and belonging to that team amounts to being a “loser” (Bernache-Assollant et al., 2018). In their paper, Rees et al. (2015) aptly illustrated this point by citing the mobility of hockey players who were members of the last place team in a championship league (Lalonde, 1992) and by demonstrating how this strategy contributes to the vitality of the professional transfer market.

Different authors have also shown that an individual may interact with others without being influenced by social comparison and categorisation (Tajfel and Turner, 1979; Haslam, 2004). Accordingly, Turner et al. (1987) asserted that an individual may also “come back” to his or her personal identity when a social identity threatens his or her self-image. Self-categorization theory (Turner et al., 1987) conceives identity processes as different identity levels, also considered as different levels of self-abstraction. Thus, a player who may not benefit from comparison with other individuals might migrate to a social identity at a higher level of self-abstraction, such as “an athlete,” which considers all people as similar in the sports context. But if the player thinks it would be more valuable to be recognized as a member of a team (because, for example, the team is at the top of the table in its league and ranked first in media polls), he or she may self-categorize at a collective identity level, which is a social identity existing in opposition to other groups (for example, the lower ranked teams). Finally, if the player considers that it would be better to compare to other individuals because of his or her higher skills, he or she may make personal identity more salient. Accordingly, during competition, identity processes may influence an athlete’s behavior by respecting the collective team strategy when he or she feels like a member of the group, or playing individualistically when he or she strives to demonstrate individual superiority. All these influential theoretical perspectives address the issue of humans’ behavior in groups, and most often, in opposition with other groups (Brown, 2000).

These important theories recognized from their early beginnings that social identity includes an affective dimension. Indeed, Tajfel and Turner (1979) explained that social identity is part of the self-concept that derives from the knowledge of, and emotional attachment to the group. One of the first attempts to associate social identity with emotions was that by Smith (1993). Smith (1993) highlighted the limitations to investigating emotions in groups that accrued because appraisal theories of emotions were cast in purely individualistic terms, this conclusion being well established in the sport literature (e.g., Hanin, 2007; Campo et al., 2012). As a consequence, he combined social identity and appraisal theories, leading later to the creation of Intergroup emotion theory (IET; Mackie et al., 2000; Smith and Mackie, 2008).

Intergroup emotion theory considers social identity as influencing the appraisal process out of which emotions arise. Synthetically, appraisal theories consider that emotions arise from individuals’ cognitions considering the person-environment transaction (Lazarus, 1999; Scherer, 2009; Scherer and Moors, 2019). In the emotion literature, opposing views exist on the number and the identity of the variables that play a role in the appraisal process (Moors et al., 2013). Nevertheless, different components of appraisal process are commonly shared among appraisal theorists such as goal-conduciveness (Lazarus, 1991; Scherer, 2004; Frijda, 2007). Most of them also consider others components influencing the person-environment transaction, such as certainty, agency (degree of responsibility in the situation that leads to a blame or credit) and coping potential (Moors et al., 2013). In sum, emotions derive from what is at stake for the individual, and are the perceived resources and the degree of responsibility in self and others. Specifically, IET argues that, although personal appraisal of events produces individual emotions that can influence behaviors, group membership and identity processes produce appraisals about events based on group priorities that lead to identity-based emotions (called intergroup emotions in IET; Smith and Mackie, 2010). In that respect, Yzerbyt et al. (2006, p. 176) proposed that “individuals carry out a cognitive evaluation of the situation they face bearing in mind that they are group members and not just unique individuals.” Although Yzerbyt’s approach and IET differ in some conceptual regards, numerous studies support the general idea that modifying social identity is associated with changes in appraisal processes which, in turn, change emotional states leading to the experience of identity-based emotions influencing intergroup behaviors (e.g., Mackie et al., 2000; Yzerbyt et al., 2006; Ray et al., 2008). For illustrations, a perception of ingroup threat and a high coping potential would lead to experience identity-based anger predicting confrontation and aggression toward the outgroup (e.g., Mackie et al., 2000; Gordijn et al., 2006). Also, identity-based guilt is experienced when people appraise the ingroup as having caused, leading to suppression of aggression, repairs and reparations (Doosje et al., 1998; Iyer et al., 2003). A last example may be found in more positive identity-based emotions, such as ‘respect’ that arises from an appraisal of the outgroup as competent and moral, such emotions predicting trust, interaction and forgiveness (Tam et al., 2009; Leonard et al., 2011; Seger et al., 2016). At the present time, while a substantive knowledge on identity-based emotions already exists in the field of social psychology in general, it is now of interest to address what is known within the context of sport in particular.

## Review of Existing Literature on Identity-Based Emotions and Sport

It is recognized that the context of sport implies multiple identities that may be salient during competition (Tamminen et al., 2016). For instance, during the *haka*, it could be supposed that a player from the New Zealand team would experience different emotions if he self-categorized as just a rugby player compared to as an “All Black.” And what drives players’ emotions when, for instance, their club merges with a former rival club, or in situations wherein players are transferred to opponent clubs and thus players once club rivals end up being teammates? Especially in the light of the professionalization of team sports, which often makes club belonging chronically salient, but also personally rewards individual performance at the expense of team goals, understanding the emotional consequences of players moving back and forth between personal and social identities may be of great interest.

Thus, to comprehensively represent research investigating identity-based emotions in sport performance settings, we undertook an exhaustive search of the literature to locate published work relevant to group level emotions in sport, using databases including Academic Search Premier, PsycINFO, PsycARTICLES, PsycEXTRA, Psychology and Behavioral Sciences Collection, and SPORTDiscus. As a first step, to locate papers published in the field of sport psychology, we used the keyword *sport*, crossed with the keywords *group-based emotions*, *intergroup emotions*, and *collective emotions*. In a second step, we combined the keyword *sport* with that of *emotions* and those of *social identity*, *group*, and *team*. It is commonly recognized that narrative reviews do not use to list the types of databases and the inclusion criteria (e.g., Cipriani and Geddes, 2003; Collins and Fauser, 2005). However, providing some key elements about the search strategy allows the readers to better judge the transparency of the work (Collins and Fauser, 2005). Moreover, this allowed us to put forward a first element in gaging the state of advancement of knowledge about identity-based emotions in sport. Indeed, one immediate evidence was the different number of outputs given the different combinations of keywords. That is, the combination *sport* x *emotions* provided 1915 papers; that of *sport* x *social identity* provided 749 records, whereas a combination of *sport* x *social identity* x *emotions* provided only 13 records. To provide some context, it is also interesting to note that the use of the keyword *intergroup emotions* or *group-based (group based) emotions* provided a total of 1091 records. Taken together, these first bibliometric comparisons clearly demonstrate a gap in the existing knowledge about the topic of identity based-emotions and its applicability in the field of sport. In addition, these numbers suggest that, despite the growing interest in social identity processes in sport, and the substantial efforts to understand emotions in this setting, the study of identity processes on athletes’ emotions seems to be only in its early days.

The relationship between emotions and social identity in the field of sport was first investigated using the Athletic Identity Measurement Scale (Brewer et al., 1993b), extending Brewer et al. (1993a), including very early on an affective dimension of social identity. Notably, the use of this questionnaire in a study of young swimmers showed that, as athletes, being distanced from the competition due to an injury meant “they would become very depressed” (Martin et al., 1995, p. 120). Similarly, some research used a tripartite model (Cameron, 2004) conceptualizing social identity as including centrality (i.e., importance to be a group member), ingroup ties (i.e., belongingness with other group members) and ingroup affects (i.e., the positivity of feelings associated with group membership). Particularly, although Bruner and Benson (2018) recently adapted Cameron (2004) 12-item questionnaire for use in the context of sport, Bruner et al. (2014) already showed that behaviors toward teammates and opponents was influenced by the affective dimension of the tripartite model of social identity. However, feeling emotions about belonging to the group (emotions toward being a group member) is quite different from identity-based emotions, that is, feeling an emotion as a group member and on behalf of what is at stake for the group (Mackie and Smith, 2018). For example, a player may feel happy about being a member of a top-level team, perhaps because membership provides evidence of self competence. That is, he or she experiences such individual emotions about the fact of being part of this reputed team. But the same player might also feel ashamed as a function of belonging to this team that is seen as usually demonstrating antisocial behaviors to win competitions, and so, feeling an identity-based guilt about the fact that his or her team behaved wrongly (Kuppens and Yzerbyt, 2012; Mackie et al., 2017).

A first attempt to examine associations between emotions and social cognitions in sport may be found in Levine and Reicher (1996) study showing that social identity influenced perceived stress engendered by injury. Building on this pioneering study in sport, other researchers have shown relationships between group cohesion variables and affective states. For example, Henderson et al. (1998) found associations between group-integration, and moods and perceived stress. Terry et al. (2000) provided evidence that individual attraction to the group and individual’s feeling of group integration predicted mood among 415 rugby players, rowers, and netballers. In a similar vein, Lowther and Lane (2002) found that variations in soccer players’ perception of cohesion was associated with changes in positive moods during the season, while a more recent study showed that individual goal relevance (primary appraisal) was influenced by group belonging processes such as an individual’s attraction to the group (Wolf et al., 2015).

Uphill and Jones’ (2007) finding that athletes’ perceptions of belonging to an ‘elite’ team influenced pride seem, however, to be the first suggestion in the literature of the existence of identity-based emotions in sport. Furthermore, it should be acknowledged that Tamminen et al. (2016), using qualitative interviews, were the first to explicitly show that participants reported the experience of emotions as a function of their social identity as athletes or team members. Recently, identity-based emotions in sport have been investigated through a series of studies by Campo et al. (2019b). One of these studies showed that the level of social identity determined the valence of emotions and individual versus group performance among elite rugby union players (Campo et al., 2019b). In addition, the authors found that social identity helped players be protected against a bias in evaluating team-referent emotions. Specifically, when personal identity was salient, the players’ emotional (negative) state influenced their appraisal of their team’s affectivity (i.e., team-referent emotions). Ultimately, this negatively influenced individual and team performances. In line with this study exploring the effect of personal versus social identities, Campo et al. (2019a) investigated the effects of two other levels of self-abstraction by comparing the emotional effects of self-categorisation to the club with those of self-categorisation to the sport, the latter social identity considered as a more inclusive social identification to a superordinate group (i.e., a higher level of self-abstraction). Their findings showed that by considering the target (ingroup or outgroup) and the identity levels, different identity-based emotions were experienced before competition among athletes. Most importantly, they support the conceptual notion of multiple identities (Deschamps and Doise, 1979; Roccas and Brewer, 2002) by providing evidence that emotions in sport depend on the interaction of several sport-related social identities. These results complemented Tamminen et al. (2016) findings of emotional conflicts due to the concomitant effects of personal and social identities. In a similar vein, with an innovative methodological approach that allowed to continuously capture the over-time variability of emotions (pleasant and unpleasant), identity levels (personal and social) and performances (individual and collective) experienced during volleyball games, Campo et al. (2018) analyzed the influences of personal and social identities. However, their results showed only a partial independence between both these levels of self-abstraction. Also, their findings highlighted that, when both personal and social identities were considered, only personal identity influenced (negatively) athletes’ emotions during competition.

Taken together, this body of research provides evidence of the influence of identity processes on emotions during competition. Nevertheless, in the light of these few existing studies, one may say that this subject is still in its infancy and that it warrants further investigation.

## Future Developments

In their review, Rees et al. (2015, p. 1091) stated that:

“until relatively recently, the potential usefulness of the social identity approach had been largely neglected in sport research. One advantage of this is that, within sport, the insights the approach affords are both novel and fresh. Accordingly, they offer opportunities to embrace a powerful new paradigm for understanding sport-related behavior.”

Along the lines suggested in our narrative review, we believe this paradigm also offers an incredible opportunity to understand emotional experiences in sport.

As we noted, several studies have shown the effect of different levels of self-abstraction on athletes’ emotions (e.g., Tamminen et al., 2016; Campo et al., 2018, 2019a,b). Moreover, it has been found that multiple sport-related social identities should be considered as autonomous constructs that can be interrelated and, sequentially or simultaneously, important to one’s self-concept (Crisp and Hewstone, 2007; Tamminen et al., 2016; Campo et al., 2018, 2019a). These findings echo different models that have been proposed to understand the complexity of the intertwinement of structural cognitive features of multiple social identities, such as intersectionality (Crenshaw, 1989), cross-categorisation (Deschamps and Doise, 1979; Crisp et al., 2002), or Social identity complexity (SIC; Roccas and Brewer, 2002). Therefore, for instance, when Levine and Reicher (1996) showed that sport students’ appraisals differed according to their social identity in terms of either “student” or “gender identity,” it may also be noted that the authors found significant effects of interaction between both identities. Similarly, Campo et al. (2019b) findings might well have been different if they had not considered the concomitant effects of both personal and social identities.

Therefore, we believe that researchers should address such crucial challenge by investigating the combination of different social identities, as well as the relative levels of personal and social identities, activated synchronously. Roccas and Brewer (2002) proposed to investigate this complexity through a continuum suggesting four types of intertwinements. The least complex, called *intersection representation*, amalgamates different social identities into an exclusive one, such as women rugby player. *Dominance* is a second combination involving the domination of one social identity on the others – which may or may not lead to differences from the dominant identity activated alone). The third level of complexity, labeled *compartmentalization*, implies that several social identities are important for the individual’s overall self-concept but are differentiated rather than merged, and are highly influenced by the social context. Lastly, *merger* is considered by the Social Identity Complexity model as the most complex configuration as the person recognizes sharing non-convergent social identities. Whichever the approach used, in an applied context such as that of sport, considering the intertwinements among different identity levels as well as among different social identities will help to better understand the complexity of athletes’ emotional experience, and thus provide more adapted methodological perspectives to investigate identity-based emotions (Yzerbyt et al., 2006).

Studying identity process in sport has been traditionally addressed through Social Identity theory and Self-Categorization theory, and more recently by the Social Identity Approach (Haslam, 2004), which merged the two latter theories. However, in the light of the other abovementioned approaches (i.e., Deschamps and Doise, 1979; Roccas and Brewer, 2002), other existing meta-theories in social identity literature may be also of interest in the study of identity-based emotions in sport. Particularly, the importance of considering the notion of group status and power asymmetries in applied contexts has become clear (Castel and Lacassagne, 2011; Kalin and Sambanis, 2018). Thus, for instance, beyond the self-esteem hypothesis associated with traditional social identity theories, Social partition theory (SPT; Castel and Lacassagne, 2011, 2015) showed that the ingroup, according to their identity interest, may be interpreted by its members as being hierarchically superior (or inferior) to another outgroup (i.e., *statutory partition*), or as having better (or worse) values (i.e., *oppositive partition*), or just as being the only one to exist as a majority group, leading to neglect of minority groups (i.e., *community partition*). These three types of social partitioning are considered as three subdivisions of a social identity influencing identity strategies and behaviors in sport (Mangin, 2015). Thus, a leader in an statutory partition will consider that coaches are superior to players and will tend to maintain a hierarchical distance from them. In turn, to protect themselves as players, athletes may move to a community partition by considering that they are “those” who perform, neglecting the role and importance of the coach. They can also consider, through an opposite partition, that their coach is using bad methods. According to the effect of identity processes on athletes’ emotions, it is therefore easy to conceive that social partitions, and more generally, group status, may also play an important role in athletes’ identity-based emotional experience.

Applying this suggestion, we believe that two other variables should be investigated in identity-based emotions in sport. The first variable is consideration of the object of emotions when studied in the context of intergroup relations. In their typology of group-level emotions, Iyer and Leach (2008, p. 89) differentiated emotions according to the target, that is, “what the emotion is felt about, an individual, in-group, or out-group?” Interestingly, this distinction has also been made in IET studies, which showed that identity-based emotions might be more fleeting than feelings according whether they are targeted toward the ingroup or the outgroup (e.g., Ray et al., 2008). In sport, this conceptual proposition was notably supported by Campo et al. (2019a), who provided evidence that identity-based emotions differed whether they were directed to fellow teammates (in-group) or to opponents (out-group). The second variable focuses on group emotional expression norms. Emotional expression norms in a social group do not account for how individuals should feel, but more for how they must express their emotions, and especially when group identification is high (Moons et al., 2009; Leonard et al., 2011). For instance, in medical practice, it is recognized that practitioners are emotionally detached and inhibited from engaging emotionally with their patients, or that if/when they need to experience the client’s emotions, they are able to regulate their own emotional experience as needed (e.g., Pletzer et al., 2015; Kerasidou and Horn, 2016). Obviously, this does not mean that these professionals are unemotional humans but simply that, as physicians and practitioners, they do not express their negative feelings or allow such feelings to interfere with their work. Accordingly, it can be assumed that emotional expression norms in a team may influence athletes’ emotional expression and may also bias athletes’ self-reports during experimental research. Indeed, if anxiety expression is prohibited in Team X., one may conceive that, during an experiment, this group emotion norm will inhibit a member of Team X. from reporting anxiety, even if he or she was feeling it.

These latter assumptions obviously raise the question of how social identities might also influence the regulation of emotions. We have already noted some identity strategies such as engaging in social mobility, focusing on one current identity rather than another, or by self-categorizing as an individual, that can help regulate emotions. Moreover, the influence of internalized group norms about experiencing and expressing emotions as members of a team should also be considered. For instance, consider players who are only supposed to exude the confident pride that bolsters performance, even if they are actually worried about their team losing. This may especially influence team captains, tasked with regulate their teammates’ as they do not know what their teammates are actually feeling (Fransen et al., 2014), and more generally ingroup dynamics as it has been suggested that identity processes may influence interpersonal emotion regulation (Campo et al., 2017). In contrast, such internalized emotional expression group norms may avoid a dysfunctional emotional contagion that would impair collective performance (Fransen et al., 2014; Campo et al., 2019b), and lead to the use of effective communal coping strategies (Lyons et al., 1998; Thorn et al., 2004; Tamminen and Gaudreau, 2014; Leprince et al., 2018), or the use of strategies aimed at influencing opponents’ emotions (Ronglan, 2007).

More generally, this highlights a need to better understand the influence of identity-based emotions on athletes’ behaviors as identity-based emotions have been recognized as leading people to adopt some specific identity-based action tendencies such aggressing the outgroup in case of identity-based anger (e.g., Mackie et al., 2000; Gordijn et al., 2006). Therefore, studying specific action tendencies associated with discrete identity-based emotions in relation to individual and team performances appears to be a heuristic way to produce novel findings in the field of emotion-performance relationship in sport. For instance, consider a rugby player who appraises an opponent as harmful to his team, even if his ingroup has the resources to win the game. This potentially leads him to experience intense group-based anger, which predicts aggressive behaviors toward the opponents (e.g., Mackie et al., 2000; Gordijn et al., 2006). Thus, if we consider that such aggressive behaviors may lead players in team contact sports to be penalized, or worst, to be severely injured (Campo et al., 2012), the question of the influence of identity-based emotions on performance is critical for the future.

However, it should also be recognized that appraisals, like action tendencies, are not stable in time (Lowe and Ziemke, 2011); for instance, given situations may vary, especially in sport competition, alongside changes in the scoreboard. In the field of identity-based emotions, Smith and Mackie (2015) explained that “over-time variability is meaningful because people may react differently toward an outgroup member depending on their current emotional state, rather than reacting the same way at many different times” (p2). Particularly, the authors showed that dynamics of identity-based appraisal led to an over-time variability in identity-based emotions, potentially implying shifts in group identification, difficulties in intergroup interactions and shifts in behaviors toward an outgroup. The dynamics of appraisals were indirectly investigated by Campo et al. (2018) who showed a great variability of the levels of social and personal identity during volleyball games. Accordingly, future research shall consider appraisals’ over-time variability. Together with the need to explore the influence of simultaneous identities, we invite researchers to explore the dynamics of multiple appraisals occurring on the basis of multiple identities to capture the actual complexity of identity-based emotional experiences in sport.

The time is ripe to systematically investigate the role of personal and social identities on the emotional process. Most importantly, while the current review might lead us to believe in a sequential relationship highlighting an influence of identity on emotions and regulation, some studies have also highlighted how emotions may help to foster group identity (for review, see Mackie and Smith, 2018). This effect has been demonstrated in sport by Tamminen et al. (2016), who reported that emotions may reinforce social identity by communicating information about group norms and values. In that sense, Townsend et al. (2014) stated that emotional similarity may help to strengthen a social identity while other authors (e.g., Smith and Mackie, 2015; Mackie and Smith, 2018) suggest that identity-based emotions may help to improve the quality of social interactions among the ingroup members. As a consequence, one should acknowledge that, while identity processes influence the experience of identity-based emotions, the contrary is true, too. With this latter perspective in mind, one may consider the identity-based emotional process as a dynamic, and future research should investigate reciprocal influences between social emotions, regulatory processes, behaviors, and group dynamics in sport. In our view, this seems a crucial theoretical basis for the practical purpose of helping coaches and sport psychologists to optimize performance.

## Author Contributions

MC leads this narrative review. Co-authors DM and XS contributed equally to the manuscript.

## Conflict of Interest Statement

The authors declare that the research was conducted in the absence of any commercial or financial relationships that could be construed as a potential conflict of interest.

## References

[B1] Bar-Eli M. Tractinsky N. (2000). Criticality of game situations and decision making in basketball: an application of performance crisis perspective. *Psychol. Sport Exerc.* 1 27–39. 10.1016/s1469-0292(00)00005-4

[B2] Barsade S. G. (2002). The ripple effects: emotional contagion and its influence on group behavior. *Adm. Sci. Q.* 47 644–675. 10.2307/3094912

[B3] Bernache-Assollant I. Bouchet P. Kada F. (2018). On predicting the relationship between team identification and supporters’ post-game identity management strategies: the mediating roles of pride and shame. *Curr. Psychol.* 1–10. 10.1007/s12144-018-9927-2

[B4] Brewer B. W. Boin P. D. Petitpas A. J. (1993a). *Dimensions of Athletic Identity.* Paper presented at the American Psychological Association Annual Conference, Toronto, Canada.

[B5] Brewer B. W. Van Raalte J. Linder D. (1993b). Athletic identity: hercules’ muscles or achilles heel? *Int. J. Sport Psychol.* 24 237–254.

[B6] Brown R. (2000). Social identity theory: past achievements, current problems and future challenges. *Eur. J. Soc. Psychol.* 30 745–778. 10.1002/1099-0992(200011/12)30:6<745::AID-EJSP24>3.0.CO;2-O

[B7] Bruder M. Fischer A. Manstead A. S. R. (2014). “Social appraisal as a cause of collective emotions,” in *Collective Emotions*, eds von Scheve C. Salmela M. (Oxford: Oxford University Press), 141–155.

[B8] Bruner M. W. Benson A. J. (2018). Evaluating the psychometric properties of the Social Identity Questionnaire for Sport (SIQS). *Psychol. Sport Exerc.* 35 181–188. 10.1016/j.psychsport.2017.12.006

[B9] Bruner M. W. Boardley I. D. Côté J. (2014). Social identity and prosocial and antisocial behavior in youth sport. *Psychol. Sport Exerc.* 15 56–64. 10.1016/j.psychsport.2013.09.003 27735231

[B10] Cameron J. E. (2004). A three-factor model of social identity. *Self Identity* 3 239–262. 10.1080/13576500444000047

[B11] Campo M. Champely S. Lacassagne M.-F. Mackie D. M. Pellet J. Louvet B. (2019a). *Athletes’ Social Identity: Its Influence on Precompetitive Group-Based Emotions.* 10.1123/jsep.2018-0282 31830744

[B12] Campo M. Champely S. Louvet B. Rosnet E. Ferrand C. Pauketat J. V. T. (2019b). Group-based emotions: evidence for emotion-performance relationships in team sports. *Res. Q. Exerc. Sport* 90 54–63. 10.1080/02701367.2018.1563274 30707087

[B13] Campo M. Martinent G. Pellet J. Boulanger J. Louvet B. Nicolas M. (2018). Emotion–performance relationships in team sport: the role of personal and social identities. *Int. J. Sports Sci. Coach.* 13 629–635. 10.1177/0123456789123456

[B14] Campo M. Mellalieu S. D. Ferrand C. Martinent G. Rosnet E. (2012). Emotions in contact team-based sports: a systematic review. *Sport Psychol.* 26 62–97. 10.1123/tsp.26.1.62

[B15] Campo M. Sanchez X. Ferrand C. Rosnet E. Friesen A. Lane A. M. (2017). Interpersonal emotion regulation in team sport: mechanisms and reasons to regulate teammates’ emotions examined. *Int. J. Sport Exerc. Psychol.* 15 1–16. 10.1080/1612197X.2015.1114501

[B16] Castel P. Lacassagne M.-F. (2011). “Contrat de communication et partitions sociales,” in *Psychologie Sociale De La Communication*, eds Castel P. SaleÌs-Wuillemin E. Lacassagne M.-F. (Paris: De Boeck), 19–34.

[B17] Castel P. Lacassagne M.-F. (2015). “Theory of social partitions and identity dynamics,” in *Construction of Social Psychology*, ed. Mohan B. (Lisbon: Science Press), 93–104.

[B18] Cialdini R. B. Borden R. J. Thorne A. Walker M. R. Freeman S. Sloan R. L. (1976). Basking in reflected glory: three (football) field studies. *J. Pers. Soc. Psychol.* 34 366–375. 10.1080/17461391.2015.1135987 26783830

[B19] Cipriani A. Geddes J. (2003). Comparison of systematic and narrative reviews: the example of the atypical antipsychotics. *Epidemiol. Psichiatr. Soc.* 12 146–153. 10.1017/s1121189x00002918 14610849

[B20] Collins J. A. Fauser B. C. J. M. (2005). Balancing the strengths of systematic and narrative reviews. *Hum. Reprod. Update* 11 103–104. 10.1093/humupd/dmh058 15618290

[B21] Crenshaw K. (1989). Demarginalizing the intersection of race and sex: a black feminist critique of antidiscrimination doctrine, feminist theory and antiracist politics. *Univ. Chic. Leg. Forum* 140 139–167.

[B22] Crisp R. J. Hewstone M. Miller N. (2002). A dual-route model of crossed categorization effects. *Eur. Rev. Soc. Psychol.* 13 35–74. 10.1080/10463280240000091

[B23] Crisp R. J. Hewstone M. (2007). Multiple social categorization. *Adv. Exp. Soc. Psychol.* 39 163–254. 10.1016/S0065-2601(06)39004-1

[B24] Deschamps J. C. Doise W. (1979). “L’effet du croisement des appartenances cateègorielles [Effect of cross-categorization],” in *Expeì*riences Entre Groupes [Groups Experiences], ed. Doise W. (Berlin: De Gruyter), 293–326. 10.1515/9783110807394-019

[B25] Doosje B. Branscombe N. Spears R. Manstead A. (1998). Guilty by association: when one’s group has a negative history. *J. Pers. Soc. Psychol.* 75 872–886. 10.1037/0022-3514.75.4.872

[B102] Fischer A. H. Manstead A. S. R. (2008). “Social functions of emotion,“ in *Handbook of Emotions* 3rd Edn, eds Lewis M. Haviland-Jones J. M. Feldman Barrett L. (New York, NY: Guilford) 456–468.

[B26] Fransen K. Vanbeselaere N. De Cuyper B. Coffee P. Slater M. J. Boen F. (2014). The impact of athlete leaders on team members’ team outcome confidence: a test of mediation by team identification and collective efficacy. *Sport Psychol.* 28 347–360. 10.1123/tsp.2013-0141

[B27] Friesen A. P. Devonport T. J. Sellars C. N. Lane A. M. (2013). A narrative account of decision-making and interpersonal emotion regulation using a social-functional approach to emotions. *Int. J. Sport Exerc. Psychol.* 11 203–214. 10.1080/1612197x.2013.773664

[B28] Frijda N. H. (2007). *The Laws of Emotion.* Mahwah, NJ: Erlbaum.

[B29] Gordijn E. Yzerbyt V. Y. Wigboldus D. Dumont M. (2006). Emotional reactions to harmful intergroup behavior: the impact of being associated with the victims or the perpetrators. *Eur. J. Soc. Psychol.* 36 15–30. 10.1002/ejsp.296

[B30] Hanin Y. Hanina M. Šašek H. Kobilšek A. (2016). Emotion-centered and action-centered coping in elite sport: task execution design approach. *Int. J. Sports Sci. Coach.* 11 566–588. 10.1177/1747954116654782

[B31] Hanin Y. L. (2007). “Emotions in sport: current issues and perspectives,” in *Handbook of Sport Psychology*, 3rd Edn, eds Tenenbaum G. Eklund R. C. (Hoboken, NJ: John Wiley & Sons), 31–58.

[B32] Hanin Y. L. (2016). “Les émotions et la performance expert en sport [Emotions and expert performance in sport],” in *Les É*motions En Sport et en EPS [Emotions in Sport and Physical Education], eds Campo M. Louvet B. (Bruxelles: De Boeck edition), 177–204.

[B33] Haslam S. A. (2004). *Psychology in Organizations: The Social Identity Approach*, 2nd Edn. London: Sage.

[B34] Henderson J. Bourgeois A. E. LeUnes A. Meyers M. C. (1998). Group cohesiveness, mood disturbance, and stress in female basketball players. *Small Group Res.* 29 212–225. 10.1177/1046496498292004

[B35] Hyun-Woo L. Youkyoum K. (2013). Discovering a gem: development of the group emotions model of sport fan experience. *Int. J. Appl. Sports Sci.* 25 127–146. 10.24985/ijass.2013.25.2.127

[B36] Iyer A. Leach C. (2008). Emotion in intergroup relations. *Eur. Rev. Soc. Psychol.* 19 86–125. 10.1080/10463280802079738

[B37] Iyer A. Leach C. W. Crosby F. J. (2003). White guilt and racial compensation: the benefits and limits of self-focus. *Pers. Soc. Psychol. Bull.* 29 117–129. 10.1177/0146167202238377 15272965

[B38] Jones M. V. Coffee P. Sheffield D. Yanguűez M. Barker J. B. (2012). Just a game? Changes in English and Spanish soccer fans’ emotions in the 2010 World Cup. *Psychol. Sport Exerc.* 13 162–169. 10.1016/j.psychsport.2011.10.008

[B39] Kalin M. Sambanis N. (2018). How to think about social identity. *Annu. Rev. Polit. Sci.* 21 239–257. 10.1146/annurev-polisci-042016-024408

[B40] Keltner D. Haidt J. (1999). Social functions of emotions at four levels of analysis. *Cogn. Emot.* 13 505–521. 10.1080/026999399379168

[B41] Kerasidou A. Horn R. (2016). Making space for empathy: supporting doctors in the emotional labour of clinical care. *BMC Med. Ethics* 17:8. 10.1186/s12910-016-0091-7 26818248 PMC4728886

[B42] Kessler T. Hollbach S. (2005). Group-based emotions as determinants of ingroup identification. *J. Exp. Soc. Psychol.* 41 677–685. 10.1016/j.jesp.2005.01.001

[B43] Kuppens T. Yzerbyt V. Y. (2012). Group based emotions: the impact of social identity on appraisals, emotions and behaviors. *Basic Appl. Soc. Psychol.* 34 20–33. 10.1080/01973533.2011.637474

[B44] Lalonde R. N. (1992). The dynamics of group differentiation in the face of defeat. *Pers. Soc. Psychol. Bull.* 18 336–342. 10.1177/0146167292183010

[B45] Lazarus R. S. (1991). *Emotion and Adaptation.* New York, NY: Oxford University Press.

[B46] Lazarus R. S. (1999). *Stress and Emotion: A New Synthesis.* New York, NY: Springer.

[B47] Lazarus R. S. (2000). How emotions influence performance in competitive sports. *Sport Psychol.* 14 229–252. 10.1123/tsp.14.3.229

[B48] Leach C. W. Spears R. Branscombe N. R. Doosje B. (2003). Malicious pleasure: schadenfreude at the suffering of another group. *J. Pers. Soc. Psychol.* 84 932–943. 10.1037/0022-3514.84.5.932 12757139

[B49] Leonard D. J. Moons W. G. Mackie D. M. Smith E. R. (2011). We’re mad as hell and we’re not going to take it anymore: anger self-stereotyping and collective action. *Group Processes Intergroup Relat.* 14 99–111. 10.1177/1368430210373779

[B50] Leprince C. D’Arripe-Longueville F. Doron J. (2018). Coping in teams: exploring athletes’ communal coping strategies to deal with shared stressors. *Front. Psychol.* 9:1908. 10.3389/fpsyg.2018.01908 30356814 PMC6189515

[B51] Levine R. M. Reicher S. D. (1996). Making sense of symptoms: self-categorization and the meaning of illness and injury. *Br. J. Soc. Psychol.* 35 245–256. 10.1111/j.2044-8309.1996.tb01095.x 8689097

[B52] Lickel B. Schmader T. Curtis M. Scarnier M. Ames D. R. (2005). Vicarious shame and guilt. *Group Processes Intergroup Relat.* 8 145–157. 10.1177/1368430205051064

[B53] Lowe R. Ziemke T. (2011). The feeling of action tendencies: on the emotional regulation of goal-directed behavior. *Front. Psychol.* 2:346. 10.3389/fpsyg.2011.00346 22207854 PMC3246364

[B54] Lowther J. Lane A. (2002). Relationships between mood, cohesion and satisfaction with performance among soccer players. *Athletic Insight Online J. Sport Psychol.* 4 126–142.

[B55] Lyons R. F. Mickelson K. D. Sullivan M. J. L. Coyne J. C. (1998). Coping as a communal process. *J. Soc. Pers. Relationsh.* 15 579–605. 10.1177/0265407598155001

[B56] Mackie D. M. Devos T. Smith E. R. (2000). Intergroup emotions: explaining offensive action tendencies in an intergroup context. *J. Pers. Soc. Psychol.* 79 602–616. 10.1037/0022-3514.79.4.602 11045741

[B57] Mackie D. M. Munasinghe A. Reed M. Pauketat J. Weisman A. Smith E. R. (2017). *Emotions Towards and Emotional Ties to the Ingroup and Ingroup Members.*, Santa Barbara, CA: University of California.

[B58] Mackie D. M. Smith E. R. (1998). Intergroup relation: insights from a theoretically integrative approach. *Psychol. Rev.* 105 499–529. 10.1037/0033-295X.105.3.499 9697429

[B59] Mackie D. M. Smith E. R. (2018). Intergroup emotions theory: production, regulation, and modification of group-based emotions. *Adv. Exp. Soc. Psychol.* 58 1–69. 10.1016/bs.aesp.2018.03.001

[B60] Mangin F. (2015). *La Co-construction Des Positionnements Identitaires Des Enseignants Et Des Élèves en Eps. [The Co-construction of the Identical Positionings of Teachers and Pupils in Physical Education].* Doctoral dissertation. Retrieved from https://tel.archives-ouvertes.fr/tel-01242898/document.

[B61] Manstead A. S. R. Fischer A. H. (2001). “Social appraisal: the social world as object of and influence on appraisal processes,” in *Appraisal Processes in Emotion: Theory, Methods, Research; Appraisal Processes in Emotion: Theory, Methods, Research*, eds Scherer K. R. Schorr A. Johnstone T. (New York, NY: Oxford University Press), 221–232.

[B62] Martin J. J. Adams-Mushett C. Smith K. L. (1995). Athletic identity and sport orientation of adolescent swimmers with disabilities. *Adapt. Phys. Activ. Q.* 12 113–123. 10.1123/apaq.12.2.113

[B63] Martiny S. E. Gleibs I. H. Parks-Stamm E. J. Martiny-Huenger T. Froehlich L. Harter A.-L. (2015). Dealing with negative stereotypes in sports: the role of cognitive anxiety when multiple identities are activated in sensorimotor tasks. *J. Sport Exerc. Psychol.* 37 379–392. 10.1123/jsep.2014-0284 26442769

[B64] Moll T. Jordet G. Pepping G.-J. (2010). Emotional contagion in soccer penalty shootouts: celebration of individual success is associated with ultimate team success. *J. Sports Sci.* 28 983–992. 10.1080/02640414.2010.484068 20544488

[B65] Moons W. G. Leonard D. J. Mackie D. M. Smith E. R. (2009). I feel our pain: antecedents and consequences of emotional self-stereotyping. *J. Exp. Soc. Psychol.* 45 760–769. 10.1016/j.jesp.2009.04.016

[B66] Moors A. Ellsworth P. C. Scherer K. R. Frijda N. H. (2013). Appraisal theories of emotion: state of the art and future development. *Emot. Rev.* 5 119–124. 10.1177/1754073912468165

[B67] Nicholls A. R. Jones C. R. Polman R. C. J. Borkoles E. (2009). Acute sport-related stressors, coping, and emotion among professional rugby union players during training and matches. *Scand. J. Med. Sci. Sports* 19 113–120. 10.1111/j.1600-0838.2008.00772.x 18282223

[B68] Niedenthal P. M. Brauer M. (2012). Social functionality of human emotion. *Annu. Rev. Psychol.* 63 259–285. 10.1146/annurev.psych.121208.131605 22017377

[B69] Parkinson B. Fischer A. H. Manstead A. S. R. (2005). *Emotion in Social Relations: Cultural, Group, and Interpersonal Processes.* New York, NY: Psychology Press 10.4324/9780203644966

[B70] Peters K. Kashima Y. (2007). From social talk to social structure: how disseminating emotions with everyday social talk structures social relationships. *J. Pers. Soc. Psychol.* 93 780–797. 10.1037/e633982013-832 17983300

[B71] Pletzer J. L. Sanchez X. Scheibe S. (2015). Practicing psychotherapists are more skilled at downregulating negative emotions than other professionals. *Psychotherapy* 52 346–350. 10.1037/a0039078 25938790

[B72] Ray D. G. Mackie D. M. Rydell R. J. Smith E. R. (2008). Changing categorization of self can change emotions about outgroups. *J. Exp. Soc. Psychol.* 44 1210–1213. 10.1016/j.jesp.2008.03.014

[B73] Rees T. Haslam S. A. Coffee P. Lavallee D. (2015). A social identity approach to sport psychology: principles, practice, and prospects. *Sports Med.* 45 1083–1096. 10.1007/s40279-015-0345-4 26092187

[B74] Roccas S. Brewer M. B. (2002). Social identity complexity. *Pers. Soc. Psychol. Rev.* 6 88–106. 10.1207/S15327957PSPR0602_01

[B75] Ronglan L. T. (2007). Building and communicating collective efficacy: a season-long in-depth study of an elite sport team. *Sport Psychol.* 21 78–93. 10.1123/tsp.21.1.78

[B76] Schachter S. (1959). *The Psychology of Affiliation.* Stanford, CA: Stanford University Press.

[B77] Scherer K. R. (2004). “Feelings integrate the central representation of appraisal-driven response organization in emotion,” in *Feelings and Emotions: The Amsterdam Symposium*, eds Manstead A. S. R. Frijda N. H. Fischer A. H. (Cambridge: Cambridge University Press), 136–157. 10.1017/CBO9780511806582.009

[B78] Scherer K. R. (2009). The dynamic architecture of emotion: evidence for the component process model. *Cogn. Emot.* 23 1307–1351. 10.1080/02699930902928969

[B79] Scherer K. R. Moors A. (2019). The emotion process: event appraisal and component differentiation. *Annu. Rev. Psychol.* 70 719–745. 10.1146/annurev-psych-122216-011854 30110576

[B80] Seger C. R. Banerji I. Hee Park S. Smith E. R. Mackie D. M. (2016). Specific emotions as mediators of the effect of intergroup contact on prejudice: findings across multiple participant and target groups. *Cogn. Emot.* 31 923–936. 10.1080/02699931.2016.1182893 27206543

[B81] Smith E. R. (1993). “Social identity and social emotions: toward new conceptualizations of prejudice,” in *Affect, Cognition, and Stereotyping: Interactive Processes in Group Perception*, eds Mackie D. M. Hamilton D. L. (San Diego, CA: Academic Press), 297–315.

[B82] Smith E. R. Mackie D. M. (2008). “Intergroup emotions,” in *Handbook of Emotions*, 3rd Edn, eds Lewis M. Haviland-Jones J. M. Barrett L. F. (New York, NY: Guilford Press), 428–439.

[B83] Smith E. R. Mackie D. M. (2010). “Intergroup emotions theory,” in *Encyclopedia of Group Processes and Intergroup Relations*, eds Levine J. Hogg M. (Thousand Oaks, CA: Sage), 473–475.

[B84] Smith E. R. Mackie D. M. (2015). Dynamics of group-based emotions: insights from intergroup emotions theory. *Emot. Rev.* 7 349–354. 10.1177/1754073915590614

[B85] Smith E. R. Seger C. R. Mackie D. M. (2007). Can emotions be truly group level? evidence for four conceptual criteria. *J. Pers. Soc. Psychol.* 93 431–446. 10.1037/0022-3514.93.3.431 17723058

[B86] Sullivan G. B. (2018). Collective emotions: a case study of south African pride, euphoria and unity in the context of the 2010 FIFA world cup. *Front. Psychol.* 9:1252. 10.3389/fpsyg.2018.01252 30186191 PMC6110925

[B87] Tajfel H. (1978). *Differentiation Between Social Groups: Studies in the Social Psychology of Intergroup Relations.* Oxford: Academic Press.

[B88] Tajfel H. Turner J. C. (1979). “An integrative theory of intergroup conflict,” in *Psychology of Intergroup Relations*, eds Austin W. G. Worchel S. (Chicago: Nelson-Hall), 33–47.

[B89] Tam T. Hewstone M. Kenworthy J. Cairns E. (2009). Intergroup trust in Northern Ireland. *Pers. Soc. Psychol. Bull.* 35 45–59. 10.1177/0146167208325004 19106077

[B90] Tamminen K. A. Gaudreau P. (2014). “Coping, social support, and emotion regulation in teams,” in *Group Dynamics in Exercise and Sport Psychology: Contemporary Themes*, 2nd Edn, eds Beauchamp M. Eys M. (New York, NY: Routledge), 222–239.

[B91] Tamminen K. A. Palmateer T. M. Denton M. Sabiston C. Crocker P. R. Eys M. (2016). Exploring emotions as social phenomena among Canadian varsity athletes. *Psychol. Sport Exerc.* 27 28–38. 10.1016/j.psychsport.2016.07.010

[B92] Terry P. C. Carron A. V. Pink M. J. Lane A. M. Jones G. Hall M. (2000). Perceptions of group cohesion and mood in sport teams. *Group Dyn.* 4 234–243. 10.1037/1089-2699.4.3.244

[B93] Thomas W. E. Brown R. Easterbrook M. J. Vignoles V. L. Manzi C. D’Angelo C. (2017). Social identification in sports teams. *Pers. Soc. Psychol. Bull.* 43 508–523. 10.1177/0146167216689051 28903661

[B94] Thorn B. E. Keefe F. J. Anderson T. (2004). The communal coping model and interpersonal context: problems or process? *Pain* 110 505–507. 10.1016/j.pain.2004.05.006 15288389

[B95] Totterdell P. (2000). Catch moods and hitting runs: mood linkage and subjective performance in professional sport teams. *J. Appl. Psychol.* 85 848–859. 10.1037/0021-9010.85.6.848 11125650

[B96] Townsend S. S. M. Kim H. S. Mesquita B. (2014). Are you feeling what I’m feeling? Emotional similarity buffers stress. *Soc. Psychol. Pers. Sci.* 5 526–533. 10.1177/1948550613511499

[B97] Turner J. C. Hogg M. A. Oakes P. J. Reicher S. D. Wetherell M. S. (1987). *Rediscovering the Social Group: A Self-Categorization Theory.* Cambridge, MA: Basil Blackwell.

[B98] Uphill M. A. Jones M. V. (2007). Antecedents of emotions in elite athletes: a cognitive motivational relational perspective. *Res. Q. Exerc. Sport* 78 79–89. 10.1080/02701367.2007.10599406 17479577

[B99] von Scheve C. Ismer S. (2013). Towards a theory of collective emotions. *Emot. Rev.* 5 406–413. 10.1177/1754073913484170

[B100] Wolf S. A. Eys M. A. Sadler P. Kleinert J. (2015). Appraisal in a team context: perceptions of cohesion predict competition importance and prospects for coping. *J. Sport Exerc. Psychol.* 37 489–499. 10.1123/jsep.2014-0276 26524095

[B101] Yzerbyt V. Dumont M. Mathieu B. Gordijn E. Wigboldus D. (2006). “Social comparison and group-based emotions,” in *Social Comparison and Social Psychology: Understanding Cognition, Intergroup Relations, and Culture; Social Comparison and Social Psychology: Understanding Cognition, Intergroup Relations, and Culture*, ed. Guimond S. (New York, NY: Cambridge University Press), 174–205.

